# Serum Levels of Lipoprotein Lipase Are Increased in Patients with Inflammatory Bowel Disease

**DOI:** 10.3390/ijms24065194

**Published:** 2023-03-08

**Authors:** Orvelindo Rodríguez-Hernández, Marta Carrillo-Palau, Alejandro Hernández-Camba, Inmaculada Alonso-Abreu, Laura Ramos, Laura de Armas-Rillo, Candelaria Martín-González, Raquel López-Mejías, Miguel Á. González-Gay, Iván Ferraz-Amaro

**Affiliations:** 1 Division of Endocrinology, Hospital Universitario de Canarias, 38320 Tenerife, Spain; 2 Division of Gastroenterology, Hospital Universitario de Canarias,38320 Tenerife, Spain; 3 Division of Gastroenterology, Hospital Universitario de Nuestra Señora de la Candelaria, 38010 Tenerife, Spain; 4 Division of Health Sciences, Universidad Europea de Canarias, 38300 Tenerife, Spain; 5 Division of Internal Medicine, Hospital Universitario de Canarias, 38320 Tenerife, Spain; 6 Department of Internal Medicine, University of La Laguna (ULL), 38200 Tenerife, Spain; 7 Epidemiology, Genetics and Atherosclerosis Research Group on Systemic Inflammatory Diseases, IDIVAL, 39011 Santander, Spain; 8 Division of Rheumatology, IIS-Fundación Jiménez Díaz, 28040 Madrid, Spain; 9 Department of Medicine, University of Cantabria, 39005 Santander, Spain; 10 Cardiovascular Pathophysiology and Genomics Research Unit, School of Physiology, Faculty of Health Sciences, University of the Witwatersrand, Johannesburg 2000, South Africa; 11 Division of Rheumatology, Hospital Universitario de Canarias, 38320 Tenerife, Spain

**Keywords:** inflammatory bowel disease, lipoprotein lipase, lipid profile

## Abstract

Disruption of the lipid profile is commonly found in patients with inflammatory bowel disease (IBD). Lipoprotein lipase (LPL) is a key molecule involved in triglyceride metabolism that plays a significant role in the progression of atherosclerosis. In this study, our aim was to study whether serum LPL levels are different in IBD patients and controls and whether IBD features are related to LPL. This was a cross-sectional study that encompassed 405 individuals; 197 IBD patients with a median disease duration of 12 years and 208 age- and sex-matched controls. LPL levels and a complete lipid profile were assessed in all individuals. A multivariable analysis was performed to determine whether LPL serum levels were altered in IBD and to study their relationship with IBD characteristics. After the fully multivariable analysis, including cardiovascular risk factors and the changes in lipid profile that the disease causes itself, patients with IBD showed significantly higher levels of circulating LPL (beta coefficient 196 (95% confidence interval from 113 to 259) ng/mL, *p* < 0.001). LPL serum levels did not differ between Crohn’s disease and ulcerative colitis. However, serum C-reactive protein levels, disease duration, and the presence of an ileocolonic Crohn’s disease phenotype were found to be significantly and independently positively related to LPL. In contrast, LPL was not associated with subclinical carotid atherosclerosis. In conclusion, serum LPL levels were independently upregulated in patients with IBD. Inflammatory markers, disease duration and disease phenotype were responsible for this upregulation.

## 1. Introduction

Inflammatory bowel disease (IBD) is a chronic inflammatory disorder that encompasses two forms of intestinal inflammation: ulcerative colitis (UC) and Crohn’s disease (CD). Inflammation, carotid subclinical atherosclerosis [1], and insulin resistance [2], parameters that are all associated with cardiovascular (CV) disease, are increased in IBD. Accordingly, IBD has been associated with an increased risk of atherosclerotic CV disease, heart failure, and atrial fibrillation [3]. In addition, IBD is also associated with a state of inflammatory dyslipidemia. Although data is conflicting, the main lipid pattern reported by most studies in IBD includes a decrease in total cholesterol and low-density lipoprotein (LDL) levels and an increase in triglyceride and high-density lipoprotein (HDL) levels compared to healthy controls [4,5,6,7]. This inflammatory dyslipidemia is believed to be independently associated with more severe disease [4]. The combination of a high risk of CV disease and the low total cholesterol levels present in IBD is known as the ‘lipid paradox’ and is consistent with other inflammatory diseases such as rheumatoid arthritis and systemic lupus erythematosus [8].

Lipoprotein lipase (LPL) is an extracellular enzyme on the vascular endothelial surface that degrades circulating triglycerides in the bloodstream. LPL functions by converting triglycerides into fatty acids and glycerol. This mean that LPL will shrink chylomicrons by removing the fatty acids from triglycerides, and the fatty acids are transferred for storage in adipocytes and as fuel in skeletal muscle, while chylomicron remnants are taken up by the liver via receptor-mediated endocytosis [9]. The main inhibitors of LPL are apolipoprotein C-III (ApoC-III) and angiopoietin-like proteins. It is believed that LPL plays a significant role in the progression of atherosclerosis [10]. For this reason, therapies aimed at increasing LPL-mediated clearance of triglycerides by decreasing the activity of proteins that inhibit LPL are emerging [11,12].

In a recent piece of work, we described that patients with IBD had significantly lower serum levels of ApoC-III after a fully multivariable analysis [13]. In the present study, we assessed the serum levels of LPL in a cohort of IBD patients with a wide range of clinical manifestations. We additionally set out to study how circulating LPL relates to the lipid profile, subclinical carotid atherosclerosis, and several cardiovascular risk factors.

## 2. Results

### 2.1. Demographic, Laboratory, and Disease-Related Data

A total of 197 IBD patients with a median disease duration 12 years (interquartile range (IQR): 8–19) and 208 age- and sex-matched controls were included in this study. The demographic and disease-related characteristics of the participants are shown in Table 1. Body mass index and waist circumference were lower in the IBD patients than the controls. While there were no differences in the prevalence of smoking or obesity, the patients with IBD were less commonly hypertensive and diabetic. The CD patients mostly had the ileal and non-stricturing, non-penetrating, types of CD. The median Crohn’s disease activity index (CDAI) score was 39 (IQR 7–80), and 89% of the patients were in the asymptomatic-remission category. Likewise, the median Harvey–Bradshaw index (HBI) was 2 (IQR 0–4), and most of the patients (82%) were in the remission category of this index. Regarding UC, 52% of the patients had pancolitis, and 76% of them had a partial Mayo index (pMS) that was less than 2 points. Regarding subclinical atherosclerosis, 35% of the IBD patients were found to have carotid plaque, and the mean carotid intima media thickness (cIMT) was 644 ± standard deviation (SD), which was 137. Additional information regarding the disease-related data is shown in Table 1.

### 2.2. Relationship of Disease-Related Data to LPL

The serum levels of LPL had a median value of 368 (IQR 244–525) ng/mL. The association of the demographics and disease-related data to the circulating LPL (as the dependent variable) is shown in Table 2. In this sense, some classic CV risk factors, such as age and the presence of diabetes or current smoking, were significantly and positively related to higher serum levels of LPL. However, CD and UC did not differ with respect to LPL serum levels.

With respect to disease-related data, disease duration, C-reactive protein (CRP), and the presence of the ileocolonic pattern in CD were significantly related to higher serum levels of LPL. These relationships remained significant after the multivariable analysis. In contrast, UC’s phenotypes and disease activity, fecal calprotectin, and the use of different treatments, or the presence of carotid subclinical atherosclerosis (carotid plaque or cIMT), were not significantly associated with LPL (Table 2).

### 2.3. Multivariable Analysis of the Differences in Lipid Profiles and LPL between IBD Patients and Controls

For the purpose of this analysis, the controls were considered to be the reference variable and, therefore, the differences were shown as the effect of having IBD against being a control (Table 3). In the univariable analysis, HDL cholesterol, the ApoB:ApoA1 ratio, and LPL were significantly higher in the patients with IBD compared to the controls. In contrast, the LDL:HDL cholesterol ratio, ApoA1, atherogenic index, and Apo-CIII revealed lower levels in the patients with IBD. In the multivariable adjustment (model #1 in Table 3), all these differences between the two populations were maintained except for the atherogenic index. Remarkably, LPL persisted as being significantly upregulated in the patients with IBD compared to the controls.

Because lipid-related molecules are interrelated, we performed an additional multivariable analysis adjusting for the same variables used in model #1 plus all the lipid-related molecules that were found to have a *p* value less than 0.20 in model #1 (model #2 in Table 3). Due to collinearity, lipid molecules derived from a formula (LDL:HDL, ApoB:ApoA1 ratios, and atherogenic index) were excluded from the regression model. In this final multivariable model, LPL was found to remain higher in the IBD patients compared to the controls (beta coef. 196 (95% confidence interval of 133–259) ng/mL, *p* < 0.001).

## 3. Discussion

The present work is the first in the literature in which LPL was evaluated in a large cohort of IBD patients with a wide set of manifestations. According to our results, circulating LPL was higher in the IBD patients compared to the matched controls. Furthermore, some disease-related features were found to be associated with these higher serum LPL levels. Based on our findings, we believe that LPL may have a role in the inflammatory dyslipidemia and cardiovascular disease found in IBD patients.

There is ample evidence showing that chronic inflammation impairs and alters lipoprotein metabolism and causes a variety of changes in plasma lipid and lipoprotein concentrations. This has been widely described in diseases such as systemic lupus erythematosus [14,15] and rheumatoid arthritis [16]. In addition, LPL is known to be upregulated in patients with systemic lupus erythematosus [17]. In this sense, the CRP and SLE disease damage index showed a positive and significant relationship with LPL. These findings found in systemic lupus erythematosus are consistent with our data on IBD. Moreover, LPL has differential effects on several inflammatory pathways that are relevant in atherosclerosis. With respect to this, LPL can differentially regulate TNF-alpha and interferon-γ-mediated inflammatory cytokine signal transduction pathways in human aortic endothelial cells [18]. In addition, LPL treatment has been described to reverse tumor necrosis factor alpha and very-low-density-lipoprotein-stimulated endothelial vascular cell adhesion molecule 1 (VCAM1) induction and VCAM1 promoter responses, thus, recapitulating the effects reported with synthetic peroxisome proliferator-activated receptor (PPAR) agonists [19]. This finding has suggested a novel anti-inflammatory role for LPL.

In a previous piece of work, we described that apolipoprotein C-III is downregulated in patients with IBD [13]. Since apolipoprotein C-III is a natural inhibitor of LPL, both results, an elevation of LPL and lower levels of apolipoprotein C-III, were consistent. We think that the disease, due to its inflammatory state, can lower the levels of apolipoprotein C-III and that, secondarily, in the absence of its inhibitor, it would increase LPL. In addition, we have previously studied LPL in RA [20]. In 323 patients with RA and 246 age-matched controls, after a multivariable analysis including cardiovascular risk factors, statin use, and lipid profile changes caused by the disease itself, RA patients showed lower circulating LPL levels [20]. This report supports the fact that LPL may be disrupted in inflammatory diseases.

High triglycerides levels are commonly elevated in patients with IBD compared to healthy controls, and this increase independently associates with more severe disease [4]. In our study, triglycerides levels were higher in the patients compared to the controls. However, the differences did not reach statistical significance. We think that this was due to the fact that most of the patients in our series presented remission or low clinical activity. However, elevation of LPL generally causes levels of triglycerides to drop. For this reason, the upregulation of LPL in IBD found in our work was not entirely compatible with the elevation of triglycerides that the disease usually expresses. In this sense, it is possible that the elevation of LPL is a primary consequence of the inflammation of the disease, which in turn, for unknown reasons, would not lead to a decrease in triglyceride levels.

Over the past years, LPL has attracted significant pharmacological attention as a target in the management of plasma lipid levels and, eventually, CV disease [21]. In this sense, different drugs that attempt to target LPL inhibitors, angiopoietin-like proteins and apolipoprotein C-III, have been developed. For example, evinacumab and vupanorsen (angiopoietin-like protein 3 inhibitors) and olezarsen (an antisense oligonucleotide-targeting apolipoprotein C-III) have shown efficacy in reducing LDL cholesterol [22,23], and studies are being carried out to define their effect in CV disease and CV events. However, in our cross-sectional study, we did not find a relationship between LPL and subclinical carotid atherosclerosis. Large-scale prospective cohort studies aimed at determining the relationship between serum LPL concentrations and CV disease in IBD may help to resolve this question. Similarly, drugs directed against LPL in IBD patients may have a future role in the management of the accelerated CV disease of this population.

We acknowledge the limitation that we measured the LPL serum mass and not its activity. However, although serum LPL is catalytically inactive, its mass reflects the level of systemic LPL biosynthesis, and there is an excellent correlation between mass and LPL activity, as reported elsewhere [24]. Furthermore, the cross-sectional design of our study did not allow us to infer causality. In our work, patients and controls taking statins and systemic steroids were allowed to participate. In the univariable analysis, neither one nor the other were related to LPL, and the use of statins did not differ between the patients and controls. For these reasons, it can be ruled out that the use of both drugs may have influenced our results.

In conclusion, LPL serum levels were higher in patients with IBD compared to matched controls. This was not a consequence of other changes in the lipid profile caused by the disease. Future studies are needed to clarify the implication that these findings may have in the accelerated atherosclerosis presented by patients with IBD.

## 4. Materials and Methods

### 4.1. Study Participants

This was a cross-sectional study that included 197 patients with IBD and 208 sex- and age-matched controls. All IBD patients were ≥18 years old, had an IBD diagnosis ≥ 1 year, based upon clinical, endoscopic, and histological criteria, and were periodically followed-up at inflammatory bowel disease units of two tertiary hospitals. Patients and controls taking statins and systemic steroids were allowed to participate in the study. Controls were recruited by general practitioners in primary health centers. Controls with a family history of any inflammatory bowel disease or other autoimmune disorder were excluded. Exclusion criteria for both patients and controls was an established CV disease, a glomerular filtration rate < 60 mL/min/1.73 m^2^, a history of cancer, the presence of any other chronic inflammatory disease, evidence of active infection, or any condition or pharmacological treatment different from statins that could influence lipids.

### 4.2. Data Collection, Laboratory Assessments, and Carotid Ultrasound Assessment

Surveys in IBD patients and controls were performed to assess CV risk factors and medication. Hypertension was defined as a systolic or a diastolic blood pressure higher than 140 and 90 mmHg, respectively. Standard techniques were used to measure high-sensitivity C-reactive protein (CRP) and fecal calprotectin. Disease activity in CD was assessed through the Crohn’s disease activity index (CDAI) and the Harvey–Bradshaw index (HBI) [25]. CDAI was split into asymptomatic remission (0 to 149 points), mildly to moderately active (150 to 220), moderately to severely active (221 to 450 points), and severely active to fulminant disease (451 to 1100 points) categories, as previously described [26]. Similarly, HBI was categorized as remission (0 to 4 points), mildly active disease (5 to 7 points), moderately active disease (8 to 16 points), and severely active disease (17 to 100 points) [25]. Disease activity in UC was calculated through the partial Mayo clinic score (pMS) [27]. Serum LPL mass was measured using a sensitive sandwich-enzyme-linked immunosorbent assay (ELISA) (Biomatik, Cambridge, ON, Canada). The assay sensitivity (minimum detectable concentration) for LPL was 0.58 ng/mL. Precision was estimated as an inter-assay coefficient of variability < 15% and an intra-assay coefficient of variability < 10%. For the detection of Apo-CIII, an ELISA kit was used (Elabscience, TX, USA). No significant cross-reactivity or interference between human Apo-CIII and analogues was observed with this kit. Both intra- and inter-coefficients of variability were <10% for this assay. Cholesterol, triglycerides, and HDL cholesterol were measured using an enzymatic colorimetric assay. LDL cholesterol was calculated using the Friedewald formula.

A carotid ultrasound examination was performed in IBD patients to assess carotid intima media thickness (cIMT) in the common carotid artery and to identify focal plaques in the extracranial carotid tree in patients with IBD. A commercially available scanner, EsaoteMylab 70 (Genoa, Italy), equipped with a 7–12 MHz linear transducer and an automated software-guided radiofrequency technique, Quality Intima Media Thickness in real time (QIMT, Esaote, Maastricht, Holland), was used for this purpose. As previously reported [28] based on the Mannheim consensus, plaque criteria in the accessible extracranial carotid tree (common carotid artery, bulb, and internal carotid artery) were defined as follows: a focal protrusion in the lumen measuring at least cIMT > 1.5 mm, a protrusion at least 50% greater than the surrounding cIMT, or an arterial lumen encroaching > 0.5 mm [29].

### 4.3. Statistical Analysis

Continuous variables data were expressed as mean ± standard deviation (SD) or as a median and interquartile range (IQR) for non-normally distributed variables. Univariable differences between patients and controls were assessed through Student’s t-test, the Mann–Whitney U-test, Chi squared test or Fisher’s exact test according to the normal distribution or the number of subjects. Differences between patients and controls regarding their lipid profiles were assessed through multivariable linear regression analysis using controls as the reference variable (beta coefficients express the effect of IBD over controls). The interpretation of beta coefficients interpretation was that for every 1-unit increase in the predictor variable, the outcome variable increased by the beta coefficient value. Confounding variables in this analysis were those with a statistical *p*-value < 0.20 for those differences in traditional CV risk factors between patients and controls. To neutralize the effect of other modifications on the lipid profile, an additional multivariable analysis was constructed, adding to the model those differences in lipid-related molecules between patients and controls with a *p*-value < 0.20. The relationship of demographics and disease-related data to LPL in IBD patients was analyzed through multivariable linear regression analysis (LPL was the dependent variable). All of the analyses used a 5% two-sided significance level and were performed using the STATA software, v.17/BE (Stata Corp., College Station, TX, USA). A *p*-value < 0.05 was considered statistically significant.

## Figures and Tables

**Table 1:** Characteristics of patients with inflammatory bowel disease and controls.

	Controls	IBD Patients	
	(*n* = 208)	(*n* = 197)	*p*
Age, years	50 ± 15	49 ± 10	0.25
Female, *n* (%)	124 (59)	107 (54)	0.28
Body mass index, kg/m^2^	30 ± 3	27 ± 5	<0.001
Abdominal circumference, cm	98 ± 7	94 ± 12	<0.001
Systolic blood pressure, mmHg	125 ± 13	126 ± 19	0.45
Diastolic blood pressure, mmHg	81 ± 5	74 ± 11	<0.001
Cardiovascular co-morbidity			
Smoking, *n* (%)	45 (22)	39 (20)	0.65
Diabetes, *n* (%)	29 (14)	11 (6)	0.004
Hypertension, *n* (%)	63 (30)	35 (18)	0.003
Obesity, *n* (%)	57 (27)	55 (28)	0.91
Statins, *n* (%)	47 (23)	21 (11)	0.001
IBD-related data			
Crohn’s disease, *n* (%)		130 (66)	
Ulcerative colitis, *n* (%)		67 (34)	
CRP, mg/L	2.0 (1.0–4.8)	1.8 (0.9–3.8)	0.30
Disease duration since diagnosis, years		12 (8–19)	
Crohn’s-disease-related data, *n* (%)			
A1 below 16 years		19 (15)	
A2 between 17 and 40 years		81 (62)	
A3 above 40 years		27 (21)	
L1 ileal		56 (43)	
L2 colonic		23 (18)	
L3 ileocolonic		51 (39)	
L4 isolated upper disease		11 (8)	
B1 non-stricturing, non-penetrating		73 (56)	
B2 stricturing		46 (35)	
B3 penetrating		14 (11)	
CDAI score		39 (7–80)	
Asymptomatic remission		116 (89)	
Mildly to moderately active Crohn’s disease		10 (8)	
Moderately to severely active Crohn’s disease		3 (2)	
Severely active to fulminant disease		0 (0)	
Harvey–Bradshaw Index		2 (0–4)	
Clinical remission		106 (82)	
Mildly active disease		14 (11)	
Moderately active disease		8 (6)	
Severely active disease		1 (1)	
Ulcerative-colitis-related data, *n* (%)			
Proctosigmoiditis		7 (10)	
Left-sided colitis		23 (35)	
Pancolitis		34 (52)	
Partial Mayo score		1 (0–1)	
<2		52 (78)	
≥2		15 (21)	
Fecal calprotectin, μg/g		113 (30–251)	
>150		96 (49)	
≥150		71 (36)	
Perianal disease, *n* (%)		23 (12)	
Previous surgery, *n* (%)		55 (28)	
Current prednisone, *n* (%)		6 (2)	
Prednisone, mg/day		8 (5–20)	
Oral mesalazine, *n* (%)		175 (89)	
Methotrexate, *n* (%)		22 (11)	
Azathioprine, *n* (%)		61 (31)	
Anti-TNF therapy, *n* (%)		58 (29)	
Ustekinumab, *n* (%)		8 (4)	
Vedolizumab, *n* (%)		5 (3)	
Tofacitinib, *n* (%)		4 (2)	
Carotid intima media assessment			
Carotid plaque, *n* (%)		68 (35)	
bilateral, *n* (%)		35 (18)	
cIMT, microns		644 ± 137	

Data represent mean ± SD or median (interquartile range) when data were not normally distributed. BMI: body mass index; CRP: C-reactive protein; TNF: tumor necrosis factor; cIMT: carotid intima media thickness. CDAI: Crohn’s disease activity index. CDAI was categorized as 0 to 149: asymptomatic remission; 150 to 220 points: mildly to moderately active; 221 to 450 points: moderately to severely active; 451 to 1100 points: severely active to fulminant disease. Harvey–Bradshaw index was categorized as 0 to 4 points: clinical remission; 5 to 7 points: mildly active disease; 8 to 16 points: moderately active disease; 17 to 100 points: severely active disease.

**Table 2:** Relation of demographics and disease-related data to lipoprotein lipase.

		IBD Patients (*n* = 197)		
	LPL, ng/mL Beta Coef. (95% CI), *p*			
	Univariable		Adjusted	
Age, years	6 (2–11)	0.009		
Female	21 (−78–121)	0.67		
Body mass index, kg/m^2^	−0.2 (−10–10)	0.98		
Abdominal circumference, cm	2 (−1–6)	0.22		
Systolic blood pressure, mmHg	0.7 (−2–3)	0.56		
Diastolic blood pressure, mmHg	−0.5 (−5–4)	0.81		
Cardiovascular co-morbidity				
Smoking	150 (29–271)	0.016		
Diabetes	263 (52–473)	0.015		
Hypertension	127 (−1–255)	0.053		
Obesity	−6 (−120–108)	0.92		
Statins	56 (−114–225)	0.52		
IBD-related data				
Crohn’s disease	ref.			
Ulcerative colitis	−40 (−144–65)	0.45		
CRP, mg/L	13 (3–24)	0.015	15 (4–25)	0.006
Disease duration since diagnosis, years	8 (3–13)	0.002	8 (3–13)	0.001
Crohn’s-disease-related data				
A1 below 16 years	102 (−82–285)	0.27	167 (−11–344)	0.065
A2 between 17 and 40 years	ref.		ref.	
A3 above 40 years	150 (−17–317)	0.078	74 (−91–239)	0.38
L1 ileal	−43 (−168–82)	0.49		
L2 colonic	−35 (−191–120)	0.66		
L3 ileocolonic	144 (21–268)	0.023	140 (20–259)	0.022
L4 isolated upper disease	−106 (−340–129)	0.37		
B1 non-stricturing, non-penetrating	48 (−74–169)	0.44		
B2 stricturing	−73 (−200–53)	0.25		
B3 penetrating	1 (−196–199)	0.99		
CDAI score	0.04 (−0.7–0.8)	0.93		
Asymptomatic remission	ref.			
Mildly to severely active	−58 (−256–140)	0.56		
Harvey–Bradshaw Index	13 (−7–33)	0.21		
Clinical remission	ref.			
Mildly active disease	−53 (−244–139)	0.59	−38 (−219–144)	0.68
Moderately active disease	190 (−62–443)	0.14	182 (−62–426)	0.14
Ulcerative-colitis-related data				
Proctosigmoiditis	75 (−212–362)	0.60		
Left-sided colitis	ref.			
Pancolitis	27 (−158–212)	0.77		
Partial Mayo score	−8 (−66–50)	0.79		
<2	ref.			
≥2	10 (−180–201)	0.91		
Fecal calprotectin, μg/g	−0.09 (−0.2–0.04)	0.19	−0.04 (−0.2–0.08)	0.49
<150	ref.			
≥150	−11 (−124–102)	0.85		
Perianal disease	32 (−118–182)	0.68		
Previous surgery	75 (−34–184)	0.18	77 (−31–184)	0.16
Current prednisone	−191 (−473–92)	0.18	−145 (−420–129)	0.30
Prednisone, mg/day	−6 (−70–58)	0.47		
Oral mesalazine	26 (−77–129)	0.62		
Methotrexate	119 (−40–279)	0.14	60 (−100–220)	0.46
Azathioprine	−85 (−191–21)	0.12	−71 (−175–32)	0.18
Anti-TNF therapy	−21 (−128–86)	0.70		
Ustekinumab	−44 (−286–197)	0.72		
Vedolizumab	−96 (−380–187)	0.50		
Tofacitinib	−195 (−510–120)	0.22		
Carotid intima media assessment				
Carotid plaque	−7 (−112–98)	0.90		
Bilateral vs. unilateral plaque	12 (−138–162)	0.87		
cIMT, microns	0.3 (−0.03–0.7)	0.074	0.04 (−0.4–0.5)	0.87

LPL was considered to be the dependent variable. Multivariable analysis was adjusted for age, smoking, diabetes, and hypertension. BMI: body mass index; CRP: C-reactive protein; TNF: tumor necrosis factor; cIMT: carotid intima media thickness. CDAI: Crohn’s disease activity index. CDAI was categorized as 0 to 149: asymptomatic remission; 150 to 220 points: mildly to moderately active; 221 to 450 points: moderately to severely active; 451 to 1100 points: severely active to fulminant disease. Harvey–Bradshaw index was categorized as 0 to 4 points: clinical remission; 5 to 7 points: mildly active disease; 8 to 16 points: moderately active disease; 17 to 100 points: severely active disease.

**Table 3:** Multivariable analysis of the differences in lipid profile and LPL between IBD patients and controls.

	Controls	IBD Patients	Univariable	Model #1	Model #2
	(*n* = 208)	(*n* = 197)	Model	Beta Coef. (95% CI), *p*	Beta Coef. (95% CI), *p*
Lipid profile			*p*		
Cholesterol, mg/dL	198 ± 45	203 ± 49	0.35		
Triglycerides, mg/dL	144 ± 70	151 ± 89	0.38		
HDL cholesterol, mg/dL	51 ± 14	57 ± 18	0.001	7 (0.6–7), 0.018	
LDL cholesterol, mg/dL	118 ± 37	116 ± 40	0.56		
LDL:HDL cholesterol ratio	2.42 ± 0.88	2.18 ± 0.86	0.005	−0.2 (−0.4–(−0.08)), 0.005	
Non-HDL cholesterol, mg/dL	147 ± 40	146 ± 43	0.81		
Lipoprotein (A), mg/dL	38 (14–103)	26 (8–88)	0.37		
Apolipoprotein A1, mg/dL	173 ± 39	162 ± 37	0.003	−15 (−22–(−7)), <0.001	
Apolipoprotein B, mg/dL	105 ± 29	108 ± 32	0.29		
Apo B1:ApoA1 ratio	0.62 ± 0.18	0.69 ± 0.22	0.001	0.07 (0.03–0.1), <0.001	
Atherogenic index	4.05 ± 1.11	3.80 ± 1.17	0.025	−0.2 (−0.4–0.01), 0.063	
Apolipoprotein C-III, mg/dL	4.1 (2.5–6.9)	3.5 (2.8–4.4)	<0.001	−1.6 (−2.3–(−0.8)), <0.001	
Lipoprotein lipase, ng/mL	229 (181–327)	368 (244–525)	<0.001	170 (120–220), <0.001	196 (133–259), <0.001

Data represent mean ± standard deviation or median (interquartile range) when data were not normally distributed. In the regression analysis, controls were considered to be the reference variable. Lipid profile molecules were the dependent variables. HDL: high-density lipoprotein; LDL: low-density lipoprotein. Model #1: adjusted for age, smoking, diabetes, and hypertension (variables with a *p* value < 20 difference between patients and controls). Model #2: adjusted for model #1 variables + HDL cholesterol, apolipoprotein A1, and apolipoprotein C-III (variables with a *p* value < 0.20 in model #1). Due to collinearity, atherogenic index and LDL:HDL, and ApoB:ApoA1 ratios were excluded from the multivariable analyses in model #2.

## Data Availability

The data presented in this study are available on request from the corresponding author.
